# Imaging in patients with glioblastoma: A national cohort study

**DOI:** 10.1093/nop/npac048

**Published:** 2022-06-11

**Authors:** Maureen Dumba, Anna Fry, Jon Shelton, Thomas C Booth, Brynmor Jones, Haris Shuaib, Matt Williams

**Affiliations:** Department of Neuroradiology, Imperial College Healthcare NHS Trust, London, UK; Cancer Research UK, London, UK; National Cancer Registration and Analysis Service, Public Health England, London, UK; Cancer Research UK, London, UK; Department of Neuroradiology, King’s College Hospital NHS Foundation Trust, London, UK; School of Biomedical Engineering & Imaging Sciences, St Thomas’ Hospital, London, UK; Department of Neuroradiology, Imperial College Healthcare NHS Trust, London, UK; Department of Medical Physics, Guy’s & St. Thomas’ NHS Foundation Trust, London, UK; Institute of Psychiatry, Psychology & Neuroscience, King’s College London, London, UK; Department of Radiotherapy, Imperial College Healthcare NHS Trust, London, UK; Computational Oncology Lab, Institute of Global Health Innovation, Imperial College London, London, UK

**Keywords:** glioblastoma, imaging, outcomes, radiotherapy, surgery

## Abstract

**Background:**

Glioblastoma is the most common malignant brain tumor in adults and has a poor prognosis. This cohort of patients is diverse and imaging is vital to formulate treatment plans. Despite this, there is relatively little data on patterns of use of imaging and imaging workload in routine practice.

**Methods:**

We examined imaging patterns for all patients aged 15–99 years resident in England who were diagnosed with a glioblastoma between 1st January 2013 and 31st December 2014. Patients without imaging and death-certificate-only registrations were excluded.

**Results:**

The analytical cohort contained 4,307 patients. There was no significant variation in pre- or postdiagnostic imaging practice by sex or deprivation quintile. Postdiagnostic imaging practice was varied. In the group of patients who were treated most aggressively (surgical debulking and chemoradiation) and were MRI compatible, only 51% had a postoperative MRI within 72 hours of surgery. In patients undergoing surgery who subsequently received radiotherapy, only 61% had a postsurgery and preradiotherapy MRI.

**Conclusions:**

Prediagnostic imaging practice is uniform. Postdiagnostic imaging practice was variable. With increasing evidence and clearer recommendations regarding debulking surgery and planning radiotherapy imaging, the reason for this is unclear and will form the basis of further work.

Primary brain tumors are a group of rare, varied tumors, many of which carry a poor prognosis. They have the highest average number of years of life lost, and are the leading cause of cancer death in those under 40 years old.^1^ Of these, glioblastoma (WHO grade IV glioma) is the most common malignant brain tumor in adults. The optimal management of glioblastoma typically consists of maximal debulking surgery, concurrent chemoradiation, and adjuvant chemotherapy.^2,3^ Even with this treatment, median survival is 15 months. More recently, level 1 evidence has shown that adding tumor-treating fields improves outcomes, but cost-effectiveness is still being evaluated.^4^

Imaging is a key component in the management of brain tumors because presentation varies depending on the size and location of the tumor. Symptoms range from stroke mimics and seizures to more nonspecific neurology related to raised intracranial pressure such as headaches.^5,6^ As a result, a CT head is often one of the earliest investigations. For further characterization, MRI is superior; it offers better soft tissue delineation than CT^7,8^ and improved sensitivity.^9,10^ Advanced MR imaging techniques such as perfusion and permeability imaging, as well as ^1^H-magnetic resonance spectroscopy (^1^H-MRS), provide additional biological information,^7,11^ which can help to determine tumor grade at presentation, tumor transformation from low to high grade, and whether there are treatment-related effects or true disease progression after treatment.^12^

At presentation, imaging is needed to characterize the tumor in terms of probable type (in particular whether a high grade or low grade) and extent and location of disease. Typical MR sequences would include *T*_1_-weighted (pre- and post-gadolinium), *T*_2_-weighted, FLAIR, *T*_2_*-weighted (or susceptibility-weighted imaging) and diffusion-weighted imaging sequences.^10^ Stereotactic sequences are necessary for surgical planning. Additional imaging may be of benefit to assess vasculature or to assist in targeting biopsy sites by determining the regions with the most malignant biological characteristics.^10^ In patients undergoing treatment, appropriate use of MRI is part of high-quality care. Early postoperative imaging is recommended for patients undergoing debulking surgery in order to assess the extent of residual disease, both for prognostic purposes and to determine if re-operation is necessary.^13^ Imaging is essential for accurate radiotherapy planning; if the time interval is short enough then this may be the same as the postoperative scan. Radiotherapy planning requires information from both *T*_1_-weighted and *T*_2_-weighted sequences.^14^ Even if there has been minimal disruption of the tumor architecture at operation due to a limited biopsy, preoperative imaging (typically a *T*_1_-weighted postcontrast scan) is inadequate for subsequent radiotherapy planning purposes and a postprocedural MRI should be performed before chemoradiation starts.

Guidance on posttreatment imaging is variable in terms of frequency and timing. With debulking surgery, there are recommendations that an early postoperative MRI with gadolinium should be performed within 72 hours of the procedure^13^; this aims to minimize the T1 effects of the postoperative blood products that can lead to misinterpretation. Thick linear or nodular enhancement involving the surgical cavity implies a subtotal resection and has a less favorable outcome.^10,15,16^ It has been suggested that patients with a macroscopic resection of >95% have better outcomes.^15^ An MRI is also recommended at 3 months following completion of chemoradiotherapy to provide the first treatment response assessment.^10^ A 3-month moratorium has been recommended before scanning commences to stop false positive interpretation of disease progression (pseudoprogression).^13^

Much has been written on the use of advanced imaging techniques in patients with glioblastoma^7,11,12,17^ and on how imaging can refine prognosis.^18^ Although clinical trials are often held as exemplar practice, less than 10% of all patients are entered in clinical trials and there is little data on current patterns of use of imaging and imaging workload in routine practice. We have previously described the incidence and survival^19^ of patients with glioblastoma in a national incident cohort of patients in England, and have shown that neurosurgical care for patients with brain tumors in England is much more centralized than other countries.^20^ In this study, using a more detailed dataset and methodology, we present data on the patterns of imaging in patients with glioblastoma in England over a 2-year period.

## Methods

We included all patients resident in England who were diagnosed with a WHO grade IV cerebral glioma between 1st January 2013 and 31st December 2014 and who were aged between 15 and 99 years at diagnosis (Supplementary Appendix 1: inclusion criteria) using international guidance on assigning date of diagnosis.^21^ The National Cancer Registration and Analysis Service (NCRAS) in England holds data on all people diagnosed with cancer in England. We linked these patients to the Diagnostic Imaging Dataset (DID) which holds data on imaging investigations in England from April 2012.^22^ Identification of a space occupying lesion requires imaging, so we excluded patients who were registered based only on a death certificate (DCO) and those who had no CT or MRI head in the 3 months preceding diagnosis (including the date of diagnosis) in DID (Supplementary Appendix 2: imaging codes). The remaining patients were the analytical cohort.

We extracted data on demographics, radiotherapy, chemotherapy, surgery, imaging, and death. We categorized patients as having a histological diagnosis or not, and in those with a histological diagnosis, whether they had a biopsy or surgical debulking. We performed simple internal quality assurance on the data by looking at the relationship between histological diagnosis and evidence of biopsy/ surgery. Oncological treatment was defined as one of 4 mutually exclusive options: chemotherapy and radiotherapy, chemotherapy (no radiotherapy), radiotherapy (no chemotherapy), or no oncological treatment (neither chemotherapy nor radiotherapy).

We examined patterns and volume of imaging workload pre- and postdiagnosis. We defined “advanced MRI” as sequences including ^1^H-MRS, perfusion and permeability MRI, diffusion tensor MRI, or MRI functional imaging (blood oxygen level dependent) (Supplementary Appendix 2: advanced imaging codes). We defined the “MRI-diagnosis interval” as the time from each patient’s first MRI in the 3-month (13-week) period before diagnosis to the date of diagnosis (including imaging on the date of diagnosis). We defined the “total prediagnostic workload” as all imaging in the 13 weeks before diagnosis (including date of diagnosis), and the “postdiagnostic workload” as imaging taking place in the year following diagnosis. “Early postoperative imaging” was defined as imaging performed within 72 hours of surgery. We defined the “MRI-radiotherapy interval” as the shortest time interval between an MRI scan and the start of radiotherapy. Because there are a small proportion of patients who are MRI-incompatible (eg, they have metallic implantable devices or intraocular shrapnel), we defined an MRI-compatible patient subgroup, based on patients who had received at least one MRI in the dataset, and calculated all metrics in the analytical group and in the MRI-compatible subgroup. Median survival time was calculated from the date of diagnosis until either death or 10th February 2016 at which they were censored.

For each measure, we looked for evidence of variation in terms of demographic categories (age, sex, ethnicity, deprivation quintile), variation based on radiological versus histological diagnosis, debulking surgery or biopsy, and the 4 oncological treatment pathways.

All analysis was carried out in Stata 13.1.

### Ethics Approval and Consent to Participate

As this was a retrospective linkage study of routinely treated patients who were subsequently de-identified, ethical approval was not required. No individual personal data are included in this article. The study was performed in accordance with the Declaration of Helsinki.

## Results

There were 4,778 patients diagnosed with cerebral glioblastoma over the 2-year period. We excluded 66 patients as they were diagnosed based on a DCO and a further 405 who had no CT or MRI head imaging record in DID in the 3 months preceding diagnosis. This left an analytical cohort of 4,307. Demographic variables for the analytical cohort are presented in Table 1. The median age was 65 years (interquartile range [IQR] 57–73), and 61% were male. Eighty-two percent of patients had a histological diagnosis, of whom 73% (2,557/3,526) underwent debulking surgery (as opposed to biopsy). Seventeen percent of patients (735/4,307) had no record of histology, surgery, or biopsy and were diagnosed based on imaging alone. Twenty-eight patients (0.1% of the cohort) had a record indicating debulking surgery, but with no record of histology. Thirty-five percent (1,522/4,307) of patients underwent chemoradiation, 35% had no oncological treatment, and the remaining 30% had either radiotherapy alone, or chemotherapy alone (Table 1). Median survival of the 4,307 patients, including the 86% of patients who died during follow-up, was 207 days (IQR 82–434).

**Table 1.** Numbers of Patients and Proportions (out of the analytical cohort) Receiving MRI or CT, MRI Brain, CT Brain, and Both CT Brain and MRI Brain, at Any Point Surrounding Diagnosis, and in the 13 Weeks Before (or on the date of) Diagnosis

		Imaging at Any Point								Imaging in the 13 wk Before Diagnosis (91 d, inclusive, including diagnosis date)					
		Patients With MRI or CT (=analytical cohort)		MRI Brain		CT Brain		MRI and CT Brain		MRI Brain		CT Brain		MRI and CT Brain	
		N	%	N	%	N	%	N	%	N	%	N	%	N	%
Total		4,307	100	3,999	93	4,148	96	3,840	89	3,428	80	3,747	87	2,868	67
Sex	Male	2,611	61	2,440	93	2,524	97	2,353	90	2,081	80	2,276	87	1,746	67
	Female	1,696	39	1,559	92	1,624	96	1,487	88	1,347	79	1,471	87	1,122	66
Age groups	Under 50	556	13	542	97	534	96	520	94	468	84	439	79	351	63
	50–59	813	19	779	96	781	96	747	92	688	85	704	87	579	71
	60–69	1,449	34	1,370	95	1,405	97	1,326	92	1,231	85	1,266	87	1,048	72
	70–79	1,107	26	1,011	91	1,059	96	963	87	855	77	981	89	729	66
	80+	382	9	297	78	369	97	284	74	186	49	357	93	161	42
Ethnicity	White	3,983	92	3,715	93	3,843	96	3,575	90	3,191	80	3,470	87	2,678	67
	Non-White	223	5	200	90	213	96	190	85	171	77	191	86	139	62
	Unknown	101	2	84	83	92	91	75	74	66	65	86	85	51	50
Deprivation	1 (least deprived)	1,004	23	929	93	977	97	902	90	803	80	873	87	672	67
	2	1,044	24	987	95	994	95	937	90	852	82	894	86	702	67
	3	870	20	804	92	842	97	776	89	697	80	758	87	585	67
	4	792	18	728	92	751	95	687	87	615	78	693	88	516	65
	5 (most deprived)	597	14	551	92	584	98	538	90	461	77	529	89	393	66
Histology	Yes	3,526	82	3,353	95	3,411	97	3,238	92	3,058	87	3,035	86	2,567	73
	No	781	18	646	83	737	94	602	77	370	47	712	91	301	39
Surgery	Yes	2,582	60	2,481	96	2,504	97	2,403	93	2,229	86	2,210	86	1,857	72
	No	1,725	40	1,518	88	1,644	95	1,437	83	1,199	70	1,537	89	1,011	59
Biopsy	Yes	1,187	28	1,102	93	1,150	97	1,065	90	1,033	87	1,046	88	892	75
	No	3,120	72	2,897	93	2,998	96	2,775	89	2,395	77	2,701	87	1,976	63
Surgery or biopsy	Yes	3,518	82	3,348	95	3,407	97	3,237	92	3,051	87	3,031	86	2,564	73
	No	789	18	651	83	741	94	603	76	377	48	716	91	304	39
Treatment	No treatment	1,506	35	1,282	85	1,439	96	1,215	81	994	66	1,364	91	852	57
	Chemo and radio	1,522	35	1,496	98	1,471	97	1,445	95	1,328	87	1,261	83	1,067	70
	Chemo	99	2	95	96	90	91	86	87	91	92	70	71	62	63
	Radio	1,180	27	1,126	95	1,148	97	1,094	93	1,015	86	1,052	89	887	75

Ninety-three percent of the analytical cohort were MRI compatible. Eighty percent of patients had an MRI before diagnosis with 67% having both CT and MRI (Table 1). The median MRI-diagnosis interval was 11 days (IQR 5–20 days) (Table 2). Prediagnosis imaging workload showed little variation (7% of patients had > 3 MRI scans in the 13 weeks preceding diagnosis).

**Table 2.** Time Interval (days) Between First MRI, in the 13 Weeks Prior to Diagnosis, and Diagnosis

		First MRI to Diagnosis		
		N	Median (days)	IQR
Total with brain MRI in 13 wk before diagnosis (91 d, inclusive)		3,428	11	5–20
Sex	Male	2,081	11	5–20
	Female	1,347	11	5–20
Age groups	Under 50	468	9	4–20
	50–59	688	11	5–18
	60–69	1,231	12	6–20
	70–79	855	13	5–22
	80+	186	4	0–16
Ethnicity	White	3,191	11	5–20
	Non-White	171	8	4–21
	Unknown	66	9	2–20
Deprivation	1 (least)	803	11	5–20
	2	852	12	5–20
	3	697	11	5–21
	4	615	11	4–19
	5 (most)	461	11	5–20
Histology	Yes	3,058	12	6–21
	No	370	1	0–10
Surgery	Yes	2,229	12	6–20
	No	1,199	10	3–20
Biopsy	Yes	1,033	13	7–22
	No	2,395	11	4–19
Surgery or biopsy	Yes	3,051	12	6–21
	No	377	1	0–11
Treatment	No treatment	994	10	2–21
	Chemo and radio	1,328	11	5–18
	Chemo	91	17	7–31
	Radio	1,015	12	7–21

Abbreviation: IQR, interquartile range.

The median number of MRI scans in the 12 months postdiagnosis was 1 for the entire cohort, and 2 for those who had any postdiagnosis imaging. However, this varied considerably by treatment pathway. Of the 1,245 patients who underwent surgery and chemoradiation, 99% were MRI compatible and the median imaging workload for this subset in the 12 months after diagnosis was 6 MRI or CT scans (IQR 4–8). In contrast, of the 487 patients who had surgery but no oncological treatment, 89% were MRI compatible and the median workload for this subset was 2 MRI or CT scans (IQR 1–3).

Of the 2,582 patients who had surgery, 96% were MRI compatible (Table 1). Of those, 45% of patients had an early postoperative MRI (39% within 2 days) (Table 3). Early postoperative MRI was slightly more common in the 1,245 patients who had surgery and chemoradiation, where 51% had a postoperative MRI within 72 hours following surgery (Table 3).

**Table 3.** Number of Days Between Surgery and Early Postoperative MRI (within 3 days of surgery), and Total Within 14 days of Surgery

			Number of Days Between Surgery and MRI				Total Within 14 d	Total in Cohort
			0 (same day)	1	2	3		
Total		N	268	426	308	151	1,292	2,582
		%	10	16	12	6	50	
Sex	Male	N	158	268	184	94	788	1,589
		%	10	17	12	6	50	
	Female	N	110	158	124	57	504	993
		%	11	16	12	6	51	
Age groups	Under 50	N	71	78	60	23	262	446
		%	16	17	13	5	59	
	50–59	N	57	108	59	44	299	582
		%	10	19	10	8	51	
	60–69	N	89	156	114	54	468	953
		%	9	16	12	6	49	
	70–79	N	49	76	69	25	237	550
		%	9	14	13	5	43	
	80+	N	2	8	6	5	26	51
		%	4	16	12	10	51	
Ethnicity	White	N	242	402	286	136	1,195	2,409
		%	10	17	12	6	50	
	Non-White	N	18	18	19	9	72	131
		%	14	14	15	7	55	
	Unknown	N	8	6	3	6	25	42
		%	19	14	7	14	60	
Deprivation	1	N	65	99	52	36	284	618
		%	11	16	8	6	46	
	2	N	61	113	78	44	327	623
		%	10	18	13	7	52	
	3	N	43	99	73	22	261	518
		%	8	19	14	4	50	
	4	N	57	71	49	28	229	463
		%	12	15	11	6	49	
	5	N	42	44	56	21	191	360
		%	12	12	16	6	53	
Histology	Yes	N	265	423	307	148	1,280	2,557
		%	10	17	12	6	50	
Biopsy	Yes	N	15	22	8	4	59	251
		%	6	9	3	2	24	
Surgery	Yes	N	268	426	308	151	1,292	2,582
		%	10	16	12	6	50	
Treatment	No treatment	N	46	54	41	16	174	487
		%	9	11	8	3	36	
	Chemo and radio	N	137	255	158	82	710	1,245
		%	11	20	13	7	57	
	Chemo	N	3	8	5	1	19	64
		%	5	13	8	2	30	
	Radio	N	82	109	104	52	389	786
		%	10	14	13	7	49	

Of the 2,519 patients who were MRI compatible, had a histological diagnosis, and underwent radiotherapy (with or without chemotherapy), 51% underwent an MRI after the date of surgery and on or before the start of radiotherapy (Table 4).

**Table 4.** Number and Proportions of Patients Receiving a Postdiagnostic MRI in the 3 Months Prior to Starting Radiotherapy

			Total Receiving MRI Before Radiotherapy (% of those with MRI and radiotherapy)	Total of Those With Radiotherapy (of the MRI-compatible population)
Total	Total	N	1,599	2,622
		%	61	
Sex	Male	N	998	1,655
		%	60	
	Female	N	601	967
		%	62	
Age groups	Under 50	N	305	433
		%	70	
	50–59	N	380	611
		%	62	
	60–69	N	581	990
		%	59	
	70–79	N	297	533
		%	56	
	80+	N	36	55
		%	65	
Ethnicity	White	N	1,487	2,445
		%	61	
	Non-White	N	82	129
		%	64	
	Unknown	N	30	48
		%	63	
Deprivation	1	N	356	624
		%	57	
	2	N	397	656
		%	61	
	3	N	313	519
		%	60	
	4	N	292	459
		%	64	
	5	N	241	364
		%	66	
Histology	Yes	N	1,535	2,519
		%	61	
	No	N	64	103
		%	62	
Surgery	Yes	N	1,373	1,988
		%	69	
	No	N	226	634
		%	36	
Biopsy	Yes	N	243	682
		%	36	
	No	N	1,356	1,940
		%	70	
Surgery or biopsy	Yes	N	1,544	2,523
		%	61	
	No	N	55	99
		%	56	
Treatment	Chemo and Radio	N	1,007	1,496
		%	67	
	Radio	N	592	1,126
		%	53	

Seven percent of the cohort underwent one or more of the advanced MRI sequences for improved diagnostic capability (dynamic susceptibility contrast and dynamic contrast enhanced and ^1^H-MRS). Four percent of patients received an advanced MRI sequence in the 3 months prior to diagnosis, and 2% received one in the 12 months following diagnosis (Table 5). Less than 1% of the cohort received an image on the list of MRI codes of advanced MRI for surgical planning (diffusion tractography imaging and functional MRI). Less than 1% of the cohort received an image from the list of PET CT for improved diagnostic capability (^18^F-choline, ^11^C-methionine, ^18^F-fluoro-d-glucose).

**Table 5.** Count of MRI Imaging in the 12 Months After Diagnosis Among Those With the Image in the 12 Months After Diagnosis (excluding the date of diagnosis), by Sociodemographic and Treatment Variables

		Median MRI Count
Total		2
Sex	Male	2
	Female	2
Age groups	Under 50	4
	50–59	3
	60–69	2
	70–79	1
	80+	1
Ethnicity	White	2
	Non-White	1
	Unknown	1
Deprivation	1 (least deprived)	2
	2	2
	3	2
	4	2
	5 (most deprived)	2
Histology	Yes	2
	No	1
Surgery	Yes	3
	No	1
Biopsy	Yes	1
	No	2
Surgery or biopsy	Yes	2
	No	1
Treatment	No treatment	0
	Chemo and radio	4
	Chemo	2
	Radio	2

## Discussion

We have presented the first study on peridiagnostic imaging workload in a national incident brain tumor cohort. To the best of our knowledge, this work is unique. Recent reports from the United States reporting outcomes in glioblastoma use a partial national cohort^23,24^ and do not report imaging. We excluded 471 patients (10% of our incident cohort) due to DCO registrations and we do not capture patients who have their imaging entirely in the private sector or abroad. Nonetheless, our results are broadly comparable with other datasets in terms of age, sex, and survival distribution. Eighty-two percent of our cohort had a histological diagnosis, which is consistent with other data from the United Kingdom and other countries.^25^ However, there is clear evidence for low use of postoperative MRI, the low use of preradiotherapy MRI, and the very limited use of “advanced” MRI. Although these data are now somewhat old, they form a useful baseline for further work, and national-level datasets in the United Kingdom currently have a minimum of 18 months delay due to data collection, cleaning, and QA.

Prediagnostic imaging appears uniform; almost all patients will have a scan preoperatively. Details on the quality of this prediagnostic imaging are not available. Further detail on imaging parameters is available in Supplementary Appendix 3. Although there are patients who have repeated imaging episodes in the 13 weeks leading up to diagnosis, these are relatively rare. More patients have CT rather than MRI prediagnosis, consistent with clinical presentation, and there was no significant variation in pre- or postdiagnostic imaging practice by sex or deprivation quintile (Table 5). Seven percent of the MRI-compatible patients underwent biopsy or surgery without a MRI beforehand; severe presenting features that warranted emergency surgery might explain some of this.

Early postoperative imaging rates in the MRI-compatible cohort were 45%. They were slightly higher in those patients who went on to have chemoradiation (51%) and lower (31%) in those who had no oncological treatment (Table 3), suggesting some degree of postoperative decision-making based on how unwell the patients were. Nonetheless, even in the group who underwent surgical debulking, were MRI compatible, and had postoperative chemoradiation, only 51% of patients had an early postoperative MRI. This suggests that in 49% of the “fittest” group of patients, there was no attempt to objectively assess their extent of resection and it is well recognized that there is significant discordance between surgeon and MRI-based estimates of extent of resection.^16^

Similarly, only 51% of patients with a histological diagnosis who had postoperative radiotherapy had an MRI between surgery and radiotherapy. Even among those who have debulking surgery and subsequently receive radiotherapy, only 64% have a MRI performed postsurgery and preradiotherapy. Given the dependence of radiotherapy on accurate target volume definition and recent guidance,^14,26^ this is troubling.

The findings raise significant concerns for neurosurgical, oncological, and radiology practice in England. While we are unable to comment on the reasons for low rates, we do not think that they are due to missing data (patients have evidence of other imaging), sampling bias (we have a near-complete national cohort), MRI compatibility, or patients being unfit for treatment. It is well recognized that England has substantially fewer radiologists and MRI scanners than comparable countries,^27,28^ and our experience suggests that there are practical barriers to implementing early postoperative and radiotherapy planning MRI^29^. Studies such as ours, which use linked individual patient data, demonstrate the power of clinically informed detailed analyses of patterns of care; one of the strengths of our study is that we report imaging rates in clinically relevant patient populations (ie, those who are well enough to go on and receive postoperative chemoradiation). However, because of this, they are inevitably retrospective. Data QA, linkage, and analysis take time, and so we report data from 7 years ago, and we expect practice to have changed over time. Repeating such analyses should now be substantially quicker as the analytical approach has now been developed.

We have reported national peridiagnostic patterns of imaging in a national incident cohort of patients with glioblastoma. Prediagnostic imaging is expected and appropriate, but postdiagnostic imaging is variable. There appears to be a significant under use of early postoperative MRI. Furthermore, only 64% of MRI-compatible patients who underwent chemoradiation had a postoperative radiotherapy planning MRI, which seems unacceptably low and raises significant questions about national patterns of practice. Reasons for low imaging rates are not available from our data, and will form the basis of further research, as will updated analysis of more recent data.

This work uses data provided by patients and collected by the NHS as part of their care and support.

## Supplementary Material

npac048_suppl_Supplementary_Appendix_S1

npac048_suppl_Supplementary_Appendix_S2a

npac048_suppl_Supplementary_Appendix_S2b

npac048_suppl_Supplementary_Appendix_S2c

npac048_suppl_Supplementary_Appendix_S3
