# Supplementary material for: Imaging in patients with glioblastoma: A national cohort study
Source: Neurooncol Pract. 2022 Jun 11;9(6):487–95. doi: 10.1093/nop/npac048 (PMC9665056; doi:10.1093/nop/npac048)
Supplement: npac048_suppl_Supplementary_Appendix_S1 [file npac048_suppl_supplementary_appendix_s1.docx]

|  |  | **Appendix 1: Inclusion criteria** |  |  |  |  |  |
| --- | --- | --- | --- | --- | --- | --- | --- |
|  |  |  |  |  |  |  |  |
|  | A | cohort of 15-99 yr olds with GBM | 4778 |  |  |  |  |
|  |  |  |  |  |  |  |  |
|  |  | patients diagnosed via death certificate only (DCO) | 66 |  | excluded from analysis |  |  |
|  |  | pts with no MRI or CT in 13 wks before diagnosis excluded | 405 |  | excluded from analysis |  |  |
|  |  |  |  |  |  |  |  |
|  | B | total (analytical cohort) | 4307 |  | From cohort B | Earliest date | Latest date |
|  |  |  |  |  | Imaging (any) | Mar-12 | Mar-17 |
|  |  |  |  |  | Diagnosis | Jan-13 | Dec-14 |
|  |  |  |  |  | Death | Jan-13 | Feb-16 |
|  |  |  |  |  |  |  |  |
|  | C | Brain MRI-compatible population | 3999 |  | sub-group of the analytical cohort |  |  |
