# Supplementary material for: Imaging in patients with glioblastoma: A national cohort study
Source: Neurooncol Pract. 2022 Jun 11;9(6):487–95. doi: 10.1093/nop/npac048 (PMC9665056; doi:10.1093/nop/npac048)
Supplement: npac048_suppl_Supplementary_Appendix_S2a [file npac048_suppl_supplementary_appendix_s2a.docx]

| **Appendix 2: Advanced Imaging Codes** |  |  |
| --- | --- | --- |
|  |  |  |
| Preferred description | short_code NICIP | SNOMED |
| **ADVANCED MRI FOR IMPROVED DIAGNOSTIC CAPABILITY (DSC AND DCE AND 1H-MRS)** |  |  |
| MRI Head brain perfusion study | MSKPE | 419059006 |
| MRI Perfusion weighted | MPERF | 419059006 |
| MRI Head spectroscopy | MSKUS | 241672000 |
| MRI Spectroscopy | MSPEC | 241671007 |
| **ADVANCED MRI FOR SURGICAL PLANNING (DTI AND fMRI)** |  |  |
| MRI Functional imaging | MFUNC | 241603006 |
| Diffusion tensor MR cerebral nerve tract | MDTNT | 448307005 |
| **PET FOR IMPROVED DIAGNOSTIC CAPABILITY (CHOLINE, METHIONINE, FDG)** |  |  |
| NM Methionine tumour imaging local PET | NMETSO | 241445004 |
| NM Brain PET FDG | NBRAIO | 430638001 |
| NM F18 choline PET CT | NCPCH | 443234008 |
| NM C11 choline PET CT | NCC11 | 443473006 |
| NM Brain FDG PET CT | NCBRA | 443560005 |
| NM Methionine tumour PET CT | NCMTU | 444203001 |
| NM Choline brain PET F18 | NBRF18 | 931391000000101 |
