# Supplementary material for: Imaging in patients with glioblastoma: A national cohort study
Source: Neurooncol Pract. 2022 Jun 11;9(6):487–95. doi: 10.1093/nop/npac048 (PMC9665056; doi:10.1093/nop/npac048)
Supplement: npac048_suppl_Supplementary_Appendix_S2b [file npac048_suppl_supplementary_appendix_s2b.docx]

| **Appendix 2: CT codes** |  |  |
| --- | --- | --- |
| Preferred description | short_code (NICIP) | SNOMED |
| Cone beam CT guided biopsy head | KHEADB | 868911000000102 |
| Cone beam CT head | KHEAD | 868781000000106 |
| CT Base of skull | CBSSK | 432814005 |
| CT Base of skull with contrast | CBSSKC | 431619004 |
| CT Brain neck thorax Abdo and pelvis | CBNTA | 440331001 |
| CT Brain perfusion study | CSKPE | 433111008 |
| CT Brain volume | CBRVO | 443601000 |
| CT Brain volume with contrast | CBRVOC | 444037004 |
| CT Cochlea Both | CCOCB | 811141000000107 |
| CT Cochlea Left | CCOCL | 811141000000107 |
| CT Cochlea Right | CCOCR | 811141000000107 |
| CT Cone beam internal auditory meatus B | KCIAB | 866821000000102 |
| CT Cone beam internal auditory meatus Lt | KCIAL | 866821000000102 |
| CT Cone beam internal auditory meatus Rt | KCIAR | 866821000000102 |
| CT Cone beam mastoid Both | KCBMB | 866841000000109 |
| CT Cone beam mastoid Left | KCMAL | 866841000000109 |
| CT Cone beam mastoid Right | KCMAR | 866841000000109 |
| CT Cranioplasty planning | CCPP | 432904000 |
| CT Face with contrast | CFACIC | 431714002 |
| CT Guided biopsy brain | CSKUHB | 432666003 |
| CT Head | CSKUH | 408754009 |
| CT Head and neck with contrast | CHENEC | 448335009 |
| CT Head with contrast | CSKUHC | 396207002 |
| CT IAM with air contrast | CAAMBC | 824721000000105 |
| CT IAM with contrast | CIAMBC | 429857005 |
| CT IAM with intrathecal contrast | CIAMBT | 433875009 |
| CT Internal auditory meatus Both | CIAMB | 241521000 |
| CT Mastoid Both | CMSTB | 419381009 |
| CT Middle ear | CMIDE | 429926006 |
| CT Orbit with contrast Both | CORBBC | 429852004 |
| CT Petrous bones | CPTRS | 241522007 |
| CT Pituitary | CPITF | 241519005 |
| CT Pituitary with contrast | CPITFC | 430437000 |
| CT Post nasal space | CPNAS | 241527001 |
| CT Post nasal space with contrast | CPNASC | 431247005 |
| CT Posterior fossa | CPOSF | 241518002 |
| CT Radiotherapy planning scan head | CRTSKR | 429867000 |
| CT Radiotherapy planning scan head Cont | CRTSFR | 941551000000104 |
| CT Radiotherapy planning scan orbit Both | CRTOBR | 430440000 |
| CT Sinuses with contrast | CSINUC | 431492000 |
| CT Stereotaxis | CSTET | 241523002 |
| CT Temporal bones | CTEMP | 429869002 |
| CT Venogram cerebral | CVECE | 432841000 |
| CT Venogram Intracranial | CVEIC | 432842007 |
| **NM Brain FDG PET CT** | **NCBRA** | 443560005 |
| **NM Brain perfusion imaging SPECT** | **NBPIMO** | 429823002 |
| **NM Brain perfusion imaging SPECT CT** | **NCBRP** | 443170004 |
| **NM Brain scan Thallium SPECT** | **NSKTHO** | 429824008 |
| **NM Brain scan thallium SPECT CT** | **NCBRT** | 443127004 |
