# Supplementary material for: Imaging in patients with glioblastoma: A national cohort study
Source: Neurooncol Pract. 2022 Jun 11;9(6):487–95. doi: 10.1093/nop/npac048 (PMC9665056; doi:10.1093/nop/npac048)
Supplement: npac048_suppl_Supplementary_Appendix_S2c [file npac048_suppl_supplementary_appendix_s2c.docx]

| **Appendix 2: MRI codes** |  |  |
| --- | --- | --- |
| Preferred description | short_code (NICIP) | SNOMED |
| MRI Brain and cervical cord | MBRCC | 440450002 |
| MRI Brain volume | MBRVO | 504161000000105 |
| MRI Brain volume with contrast | MBRVOC | 504171000000103 |
| MRI Craniofacial | MFACI | 438834000 |
| MRI Craniofacial with contrast | MFACIC | 826751000000109 |
| MRI Diffusion weighted brain | MDWBN | 431999000 |
| MRI Diffusion weighted IAM Both | MDIMB | 911821000000101 |
| MRI Diffusion weighted IAM Left | MDIML | 911821000000101 |
| MRI Diffusion weighted IAM Right | MDIMR | 911821000000101 |
| MRI Face | MFAC | 35249003 |
| MRI Face with contrast | MFACEC | 443233002 |
| MRI Functional imaging | MFUNC | 241603006 |
| MRI Guided brain marking | MBRMA | 431402006 |
| MRI Head | MSKUH | 241601008 |
| MRI Head brain perfusion study | MSKPE | 419059006 |
| MRI Head spectroscopy | MSKUS | 241672000 |
| MRI Head stereotactic planning | MBRASR | 359071000000107 |
| MRI Head with contrast | MSKUHC | 432874000 |
| MRI IAM with contrast Both | MIAMBC | 431625000 |
| MRI Orbit with contrast Both | MORBBC | 433133005 |
| MRI Perfusion weighted | MPERF | 419059006 |
| MRI Phosphorus spectroscopy head | MPSHE | 241673005 |
| MRI Pituitary | MPITF | 241605004 |
| MRI Pituitary with contrast | MPITFC | 433132000 |
| MRI Post nasal space | MPNAS | 241610000 |
| MRI Posterior fossa | MPOFO | 241604000 |
| MRI Posterior fossa with contrast | MPOFOC | 439084004 |
| MRI Radiotherapy planning scan | MRTXPR | 228712001 |
| MRI Radiotherapy planning scan head | MRTSKR | 432022004 |
| MRI Radiotherapy planning scan orbits | MRTOBR | 430165007 |
| MRI Skull base | MSKBA | 446772005 |
| MRI Spectroscopy | MSPEC | 241671007 |
| MRI Subthalamic nucleus | MSTHN | 443516005 |
| MRI Temporal lobe | MTEMP | 447334006 |
| Diffusion tensor MR cerebral nerve tract | MDTNT | 448307005 |
| MRI Diffusion weighted | MDIFF | 440408002 |
