# Supplementary material for: Imaging in patients with glioblastoma: A national cohort study
Source: Neurooncol Pract. 2022 Jun 11;9(6):487–95. doi: 10.1093/nop/npac048 (PMC9665056; doi:10.1093/nop/npac048)
Supplement: npac048_suppl_Supplementary_Appendix_S3 [file npac048_suppl_supplementary_appendix_s3.docx]

**Appendix 3: Structural Imaging Parameters**

|  | T1W | T2W | DWI | FLAIR |
| --- | --- | --- | --- | --- |
| NHNN | 3D spoiled gradient echo sequence  TR: 8.2-8.6 ms  TE: 3.2–3.4 ms  FA: 12°  Matrix size: 256 × 256  Field of view (FOV): 220 × 220  Slice thickness: 1.2mm. | fast spin echo sequence  TR: 3000-6000 ms  TE: 80–102 ms  FA: 90°  Matrix size: 512 × 512  Field of view (FOV): 192 × 240  Slice thickness: 5mm | 2D echo-planar imaging sequence  TR: 6500-11000 ms  TE, 55–101 ms  FA: 90°  Matrix size: 256 × 256  Field of view (FOV): 240 × 240  Slice thickness: 4-5mm | 2D spin echo sequence  TR: 3000-6000 ms  TE: 88–140 ms  FA: 90°/150°  Matrix size: 512 × 512  Field of view (FOV): 220 × 220  Slice thickness: 4-5mm |
| KCH | 3D variable angle fast spin echo sequence  TR: 500-600 ms  TE: 7.2–8.9 ms  FA: 12°0  Matrix size: 256 × 256  Field of view (FOV): 250 × 250  Slice thickness: 1mm | Fast spin echo sequence  TR: 3960-7570 ms  TE: 87–99 ms  FA: 120°/150°  Matrix size: 324 × 384  Field of view (FOV): 230 × 290  Slice thickness: 5mm | 2D echo-planar imaging sequence  TR: 3500-7390 ms  TE: 61–91 ms  FA: 90°  Matrix size: 168 × 168  Field of view (FOV): 230 × 230  Slice thickness: 4-5mm | 2D spin echo sequence  TR: 5000-9000 ms  TE: 86–120 ms  FA: 90°/150°  Matrix size: 250 × 320  Field of view (FOV): 230 × 270  Slice thickness: 4-5mm. |

 Key

NHNN – National Hospital for neurology and Neurosurgery, London UK

KCH – Kings College Hospital, London UK

The table above shows structural imaging parameters used in two brain tumour protocols, each from a representative UK hospital. Up to 10% of the 31 UK neuro-oncology centres use advanced imaging routinely and up to 35% on selected cases ^29^.
